# Design and Fabrication of Interdigital Supercapacitors as Force/Acceleration Sensors

**DOI:** 10.3390/s22239268

**Published:** 2022-11-28

**Authors:** Jue Huang, Keren Dai, Yajiang Yin, Zhaorong Chen, Zheng You, Xiaofeng Wang

**Affiliations:** 1 Department of Precision Instrument, Tsinghua University, Beijing 100084, China; 2 Beijing Advanced Innovation Center for Integrated Circuits, Beijing 100084, China; 3 ZNDY of Ministerial Key Laboratory, School of Mechanical Engineering, Nanjing University of Science and Technology, Nanjing 210094, China

**Keywords:** supercapacitor, interdigital, sensing, integration

## Abstract

The integrated device for energy supply and sensing (IDESS) is a potential candidate for relieving the energy and space burdens caused by the rising integration degrees of microsystems. In this article, we propose a force sensor based on an interdigital supercapacitor (IDTSC). The capacitance and internal resistance of the IDTSC change under external loads, resulting in a transient current fluctuation at a constant bias voltage, which can be used to sense external force/acceleration. The IDTSC showed a specific energy and specific power of 4.16 Wh/kg and 22.26 W/kg (at 0.1 A/g), respectively, which could maintain an essential energy supply. According to the simulation analysis, the designed IDTSC’s current response exhibited good linearity with the external force. In addition, benefiting from its light weight and the applied gel electrolytes, the IDTSC showed good high-*g* impact sensing performance (from 9.9 × 10^3^× *g* to 3.2 × 10^4^× *g*). This work demonstrated the feasibility of realizing an integrated energy supply and force-sensing device by empowering energy storage devices with sensing capabilities.

## 1. Introduction

The integrated device for energy supply and sensing (IDESS) has recently attracted much attention [1,2,3,4]. This device has energy supply and sensing capabilities and can be used to simplify electronic systems. However, integrating multi-function sensing and long-term autonomous energy supply into one device is a challenge. One efficient method is to fabricate an IDESS with certain energy-generation materials. For example, Dewangan et al. [5] fabricated an energy-harvesting system using piezoelectric sensors, achieving a 3 V voltage output with a single sensor. Kim et al. [6,7] found that carbon nanotube yarn could be an energy-harvesting material and verified its application in self-powered sensors.

One issue related to the IDESS mentioned above was that not enough power was generated to maintain a sustainable energy supply. Hence, empowering an energy storage device with sensing capabilities represents a more practical approach. Yu et al. [8] studied the transient short-circuit effect of lithium batteries under external force and revealed the possibility of using this effect to sense high-*g* impact. According to their findings, the capacitor-type sensor is a potential candidate because of the energy-storage nature of capacitors. Typically, the capacitance of conventional capacitive sensors is only about pf [9,10,11], which hardly maintains a continuous energy supply. Fabricating such sensors based on supercapacitors is a promising solution due to their significantly higher energy density than dielectric capacitors [12], and they are increasingly used in such applications [13,14,15].

In this article, we propose a force sensor based on an interdigital supercapacitor (IDTSC). The counter electrodes of this sensor move in reverse in the out-plane direction under external force, resulting in a simultaneous decrease in the capacitance and a rise in the resistance. Changes in the capacitance and resistance synergistically diminish the current during constant voltage charging, which can be used to sense external mechanical forces. Based on the finite element simulation method, the sensing characteristics of the IDTSC and the influences of some key parameters were analyzed. Guided by the simulation analysis, we fabricated an IDTSC and verified its essential electrochemical and force-sensing performance. Moreover, the high-*g* acceleration-sensing performance of the IDTSC was tested. Our IDTSC showed force/high-*g* acceleration-sensing potential and provides a reference for developing the IDESS.

## 2. Analytical Basis

**Figure 1:**
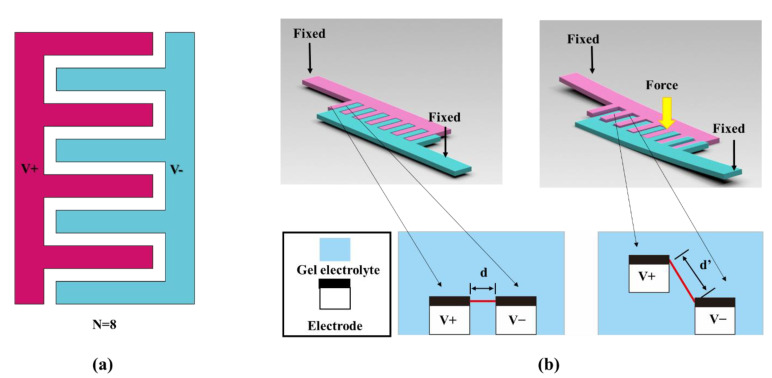
(**a**) Schematic diagram of the basic geometry of an IDTSC. (**b**) Illustration of the force-sensing process of an IDTSC.

Figure 1a shows the basic geometry of an IDTSC; the electrolyte covers all the electrodes and fills the gap, but this was ignored in the diagram. We used a gel electrolyte to replace the regular liquid electrolyte in the IDTSC to provide reliable mechanical support. This allowed the limited movement of the electrodes of the IDTSC in the out-of-plane direction. As shown in Figure 1b, once an external force is applied, the positive and negative electrodes bend and deflect, widening the gap between them. Typically, the resistance and capacitance of a supercapacitor vary under external stress/strain [16,17,18]. Our IDTSC also exhibited such coupling effects, and we attributed them to the increase in the ion transport distance in the electrolyte. On the one hand, the internal resistance increases due to the extended transport distance. On the other hand, the ion concentration in the gel electrolyte decreases because of the stretching, resulting in capacitance degradation. These changes in capacitance and resistance could synergistically diminish the current through the IDTSC during constant voltage charging. Hence, it is possible to sense the external force based on its corresponding transient current fluctuation.

In order to calculate the basic working rules and determine a design basis, an analytic model of the IDTSC was required. In the following subsections, we establish an analytical model for the resistance and capacitance changes based on the theoretical model and the finite element method.

### 2.1. Resistance Change

In our IDTSC sensor, the change in resistance resulted from the deformation of the gel electrolyte, so it was necessary to determine how the resistance of the electrolyte changed with deformation.

Typically, the resistance change of a deformed material is caused by a change in resistivity or the geometric factor [19]. Since the mobile ions dissolve in the liquid phase of the gel electrolyte, the ionic conductivity is hardly affected by deformation [20]. Therefore, we assumed that the geometric deformation dominated the resistance change. For a regular geometry, such as a cuboid or cylinder (with constant length and cross-sectional area), the resistance can be easily calculated by *R* = *ρl*/*s*. The electrolyte in a IDTSC has a complex shape, and the deformation is usually nonuniform under external forces. Therefore, it was difficult to calculate the resistance change of the electrolyte. Fortunately, the finite element method was an efficient way to calculate the resistance variation of the IDTSC under deformation, with the following equation governing the finite element simulation calculation:

(1) $$\left\{\begin{array}{l} \nabla \cdot j+\frac{dQ}{dt}=0\\ j=\sigma_{c}E\\ E=-\nabla U \end{array}\right.$$

where *j* is the surface current density, *Q* is the charge, *t* represents time, *E* is the electric field intensity, *U* is the voltage, and *σ*_c_ is the material’s electrical conductivity.

The strain of the gel electrolyte is an implicit variable that can affect the volume of the geometry, thus changing the current density and the resistance.

### 2.2. Capacitance Change

Since stress-induced capacitance changes are mainly reported in electric double-layer supercapacitors (EDLCs) [17,21,22,23], activated carbon was chosen as the electrode material of the IDTSC.

For such supercapacitors, the electric double layer in the electrode–electrolyte interface determines the capacitance. Based on Stern’s model, the double layer consists of a compact layer and a diffusion layer. The equivalent capacitance is then obtained by combining their capacitance [24,25]:

(2) $$C_{dl}=\frac{\varepsilon_{0}\varepsilon_{r}}{x_{d}+x_{s}}$$

where *ε*_0_*ε*_r_ is the permittivity; *x_s_* is the thickness of the compact layer; and *x_d_* is the Debye length of the electrolyte, which is related to the local ion concentration *c*:

(3) $$x_{d}=\sqrt{\frac{RT\varepsilon_{0}\varepsilon_{r}}{2F^{2}c}}$$

The gel electrolyte is stretched under external forces, and the ion concentration in the stretching direction is reduced. Assuming that the deformation of the electrolyte is uniform, the variation in concentration can be calculated by the strain *ε*:

(4) $$\frac{c}{c_{0}}=\frac{1}{1+\varepsilon }$$

Hence, the Debye length is related to the strain:

(5) $$\frac{x_{d}}{x_{d0}}=\sqrt{1+\varepsilon }$$

Considering that *x_d_* >> *x_s_* holds in most cases, the variation in capacitance with the strain of the gel electrolyte can be obtained as follows:

(6) $$\frac{C_{dl}}{C_{dl0}}=\frac{x_{d0}}{x_{d}}=\frac{1}{\sqrt{1+\varepsilon }}$$

### 2.3. Current Response

For most modern sensors, the output is typically an electrical signal. For example, these the authors of [7,26] used the potential fluctuation as the output signal of a supercapacitor-type sensor. Since constant current charging requires additional voltage control to prevent the supercapacitor from over-voltage, the constant voltage charging method is more common in practical application scenarios. Therefore, we chose the transient changes in the charging current as the signal of our IDTSC sensor.

**Figure 2:**
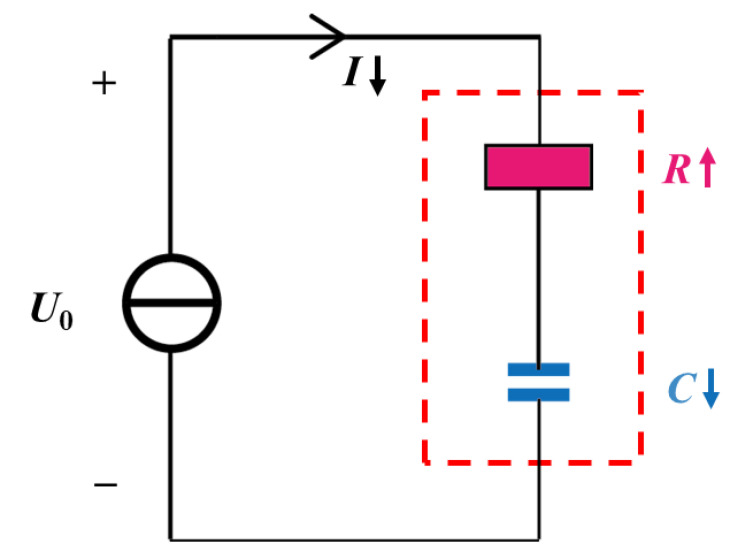
The circuit model of a supercapacitor.

As shown in Figure 2, a supercapacitor can typically be modeled as a series connection of a capacitor and a resistor [27], where the resistor and the capacitor represent the internal resistance and capacitance, respectively.

The charging current *I*_c_ of a supercapacitor at constant voltage *U*_0_ is as follows:

(7) $$I_{c}=\frac{U_{0}e^{-\frac{t}{RC}}}{R}$$

where *t* is the charging time, and *R* and *C* are the resistance and capacitance, respectively.

The current change caused by capacitance and resistance changes can be expressed as follows:

(8) $$\frac{I_{c}}{I_{c0}}=e^{\;t(\frac{1}{R_{0}C_{0}}-\frac{1}{RC})}\frac{R_{0}}{R}$$

For most common sensors, good linearity is desired. For such a sensor, the measurand can be rapidly calculated from the output signal based on simple linear fitting. Equation (8) indicates that the current change of our IDTSC was not strictly linear with variations in *R*, which may be inconvenient for measuring the external force. In addition, the charging time (*t*) also strongly influences current changes. Since the external force is generated at an arbitrary time in practical applications, a clear relationship between the current response and the external force cannot be calibrated in advance.

However, if the product of *R* and *C* remains nearly constant, i.e., *RC* is very close to *R*_0_*C*_0_, the nonlinear part of Equation (8) can be ignored, and Equation (8) is then written as follows:

(9) $$\frac{I_{c}}{I_{c0}}=\frac{R_{0}}{R}\;\mathrm{or}\;\frac{△I_{c}}{I_{c0}}=\frac{R_{0}}{R}-1$$

Equation (9) suggests that if *RC* varies slightly and *R*_0_/*R* varies linearly, the current response tends to be linear, and the influence caused by *t* is also eliminated. In a word, a decreased capacitance can compensate for the nonlinear component in Equation (8). In fact, because changes in the capacitance and the resistance of the IDTSC are in opposition, their product can remain nearly constant.

**Figure 3:**
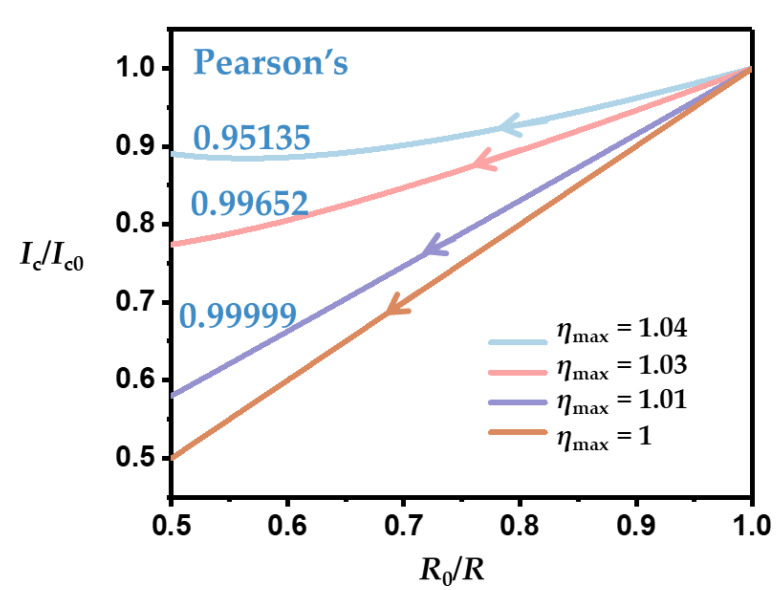
The influence of *RC* variation on *I_c_*/*I_c_*_0_ vs. *R*_0_/*R*.

We calculated the relationship between the current change and *R*_0_/*R* to show the influence of *RC*. Here, *RC* increased linearly to different maximum values [*RC*]_max_ ([*RC*]_max_ = *η*_max_·*R*_0_*C*_0_). Pearson’s coefficient is adopted to evaluate the linearity between two datasets. The closer the coefficient is to 1 or −1, the better the linearity. As *RC* changed more rapidly, the linearity of the current variation decreased (Figure 3). When *η*_max_ = 1.04, meaning that *RC* increased the most, Pearson’s coefficient was only 0.95135. Furthermore, the increased *RC* also restrained the current change, resulting in a weaker response when serving as a sensor.

**Figure 4:**
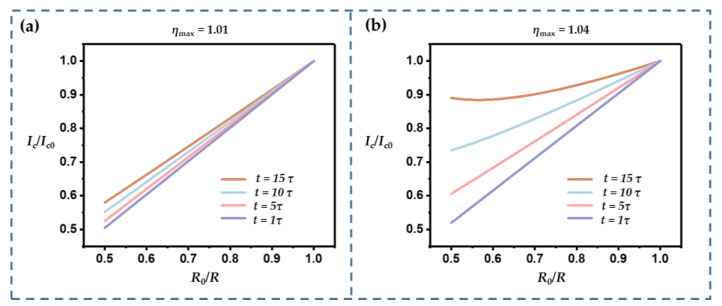
*I_c_*/*I_c_*_0_ vs. *R*_0_/*R* for different moments at which *RC* started to change: (**a**) *η*_max_ = 1.01, (**b**) *η*_max_ = 1.04.

The time variable (*t*) can strongly influence the current response. Keeping *RC* constant also helps to eliminate this time effect. Figure 4 shows the relationship between *I*_c_/*I*_c0_ and *R*_0_/*R* for different *t* and *η*_max_ values, where *t* is defined as a multiple of the time constant, *τ* (*τ* = *R*_0_*C*_0_). For each case, the time variable ranged from 1 *τ* to 5 *τ*. When *η*_max_ was small (Figure 4a), i.e., *RC* changed slightly, the curves were close and maintained good linearity. However, when *η*_max_ was larger, the curves differed significantly, and some showed nonlinearity.

The above results indicated that keeping *RC* nearly invariant and a linear variation in *R*_0_/*R* are quite important for improving the sensing performance of the IDTSC. In order to obtain a better sensing performance, a simulation optimization was implemented. The simulation focused on optimizing the linearity of the resistance variation and obtaining minimal fluctuations in *RC*.

Deformation data for the IDTSC were needed to calculate the resistance and capacitance. COMSOL Multiphysics software provides a tool for simulating deformation under different external loads. The key parameters used in the simulation are listed in Table 1.

## 3. Simulation Results

**Figure 5:**
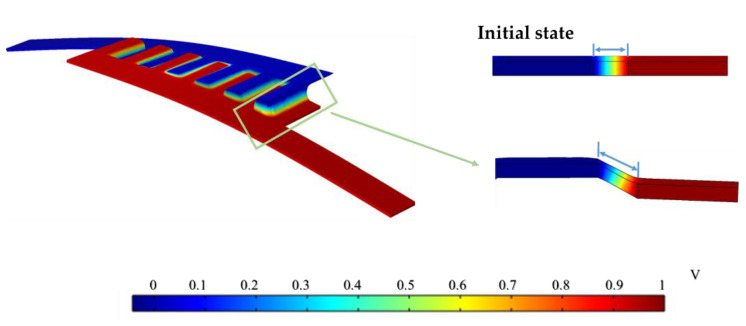
The potential distribution and deformation of the IDTSC under 1 N force.

Figure 5 shows the voltage distribution and deformation of the IDTSC under 1 N force. This simulation calculation did not involve the electrochemical physical field, so the electric field distribution does not perfectly match the real situation, especially at the electrode–electrolyte interface. However, it could determine the resistance of the conductor and the corresponding resistance change under deformation, which was sufficient for the resistance analysis in this study. The diagram on the left shows the voltage distribution of the IDTSC under deformation. The voltage changed quickly only inside the gap between electrodes, meaning the electrolyte’s resistance inside the gap contributed most of the total resistance, so its resistance fluctuation may strongly affect the total resistance of the IDTSC. The resistance of the electrolyte could be obtained by dividing the current into the voltage across the positive and negative electrodes, and the capacitance variation could be obtained by substituting the strain data into Equation (6).

**Figure 6:**
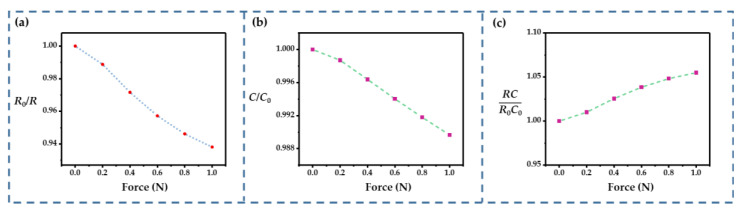
Simulation results: (**a**) *R*_0_/*R* vs. external forces; (**b**) *C*/*C*_0_ vs. external forces; (**c**) *RC*/*R*_0_*C*_0_ vs. external forces.

The capacitance and resistance change could be obtained from the simulation results. As shown in Figure 6a,b, the external force caused an increase in the resistance and a decrease in the capacitance. Both of these parameters varied almost linearly with the external force. However, *RC* reached 1.05 under an external force of 1 N (Figure 6c), which may have exacerbated the nonlinearity according to Equation (8). Hence, parameter optimization was needed to obtain a linear resistance change and more subtle *RC* change.

Since the structural and dimensional parameters are physical quantities that can be easily controlled in the processing, certain structural and dimensional parameters were investigated in the simulations.

**Figure 7:**
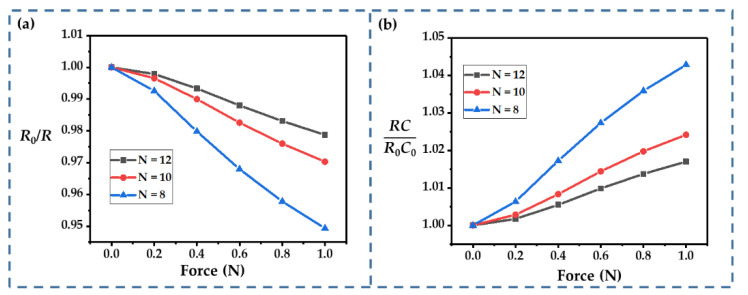
(**a**,**b**) Influence of the number of fingers on *R*_0_/*R* and *RC*/*R*_0_*C*_0_.

The number of fingers (*N*) is an important structural parameter for an interdigital-type supercapacitor; thus, we first calculated the influence of *N*, as shown in Figure 7a,b. Obviously, a smaller *N* increased the changes in *R* and *RC*: at the given ranges of *N* (from 8 to 12), *R*_0_/*R* ranged from 0.947 to 0.979, and *RC*/*R*_0_*C*_0_ ranged from 1.043 to 1.017 (at 1 N). This was probably because the subjecting of more fingers to the external force resulted in a lower average strain on each finger. According to Equation (8), increasing *N* helps to improve the linearity of the current response, because *RC* changes ony slightly. However, it may significantly increase the size of the device, so a trade-off is required in practical applications.

**Figure 8:**
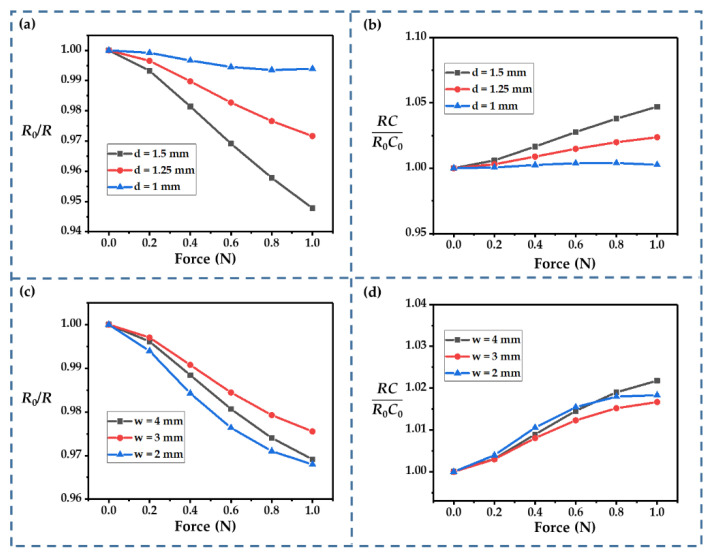
(**a**,**b**) Influence of *d* on *R*_0_/*R* and *RC*/*R*_0_*C*_0_. (**c**,**d**) Influence of *w* on *R*_0_/*R* and *RC*/*R*_0_*C*_0_.

The influence of the gap size (*d*) and the finger width (*w*) were also considered. A wider gap improved the sensitivity, which meant that the resistance changed more significantly (Figure 8a). This improvement in the sensing performance could be attributed to the strength degradation of the electrolyte film caused by the size increases. Furthermore, the relationship between the resistance and the external force showed better linearity as the gap widened. A wider gap also increased the changes in *RC* (Figure 8b). When the gap size reached 1.5 mm, *RC* was almost 1.05 times the initial value at an external force of 1 N.

For the finger width, we considered *w* values ranging from 2 to 4 mm. The value of *d* was fixed at 1.25 mm in these calculations. Among the three cases, the change in resistance was minimal when *w* = 3 mm and maximum when *w* = 2 mm. The change in resistance was in-between these two values for a width of 4 mm (Figure 8c). The nonlinearity was more significant when *w* = 2 mm, so keeping *w* at 3~4 mm may be a better choice. The finger width seemed to have a smaller effect on the change in *RC*. In all three cases, the variation in *RC* was very slight (Figure 8d).

**Figure 9:**
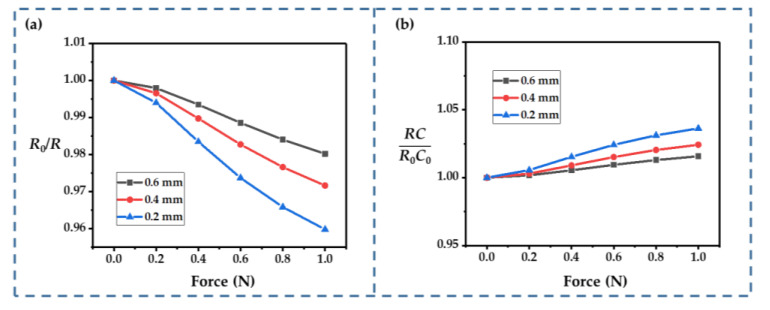
(**a**,**b**) Influence of the thickness of the electrolyte film on *R*_0_/*R* and *RC*/*R*_0_*C*_0_.

The thickness of the electrolyte is another key parameter that can influence the performance of an IDTSC. As shown in Figure 9a, changes in the resistance became more significant as the electrolyte became thinner. Typically, the film’s stiffness is positively correlated with its thickness, so a thinner film helps to boost the electrodes’ motion. As the film thickness decreased, *RC* changed more significantly (Figure 9b). Thus, though thinner films helped to enhance the current response, they exacerbated the nonlinearity introduced by the more significant change in *RC*. A thinner electrolyte film should be selected if the focus is on obtaining a more pronounced sensing response. If the goal is to measure external forces more accurately (requiring good linearity), a thicker electrolyte should be adopted.

**Figure 10:**
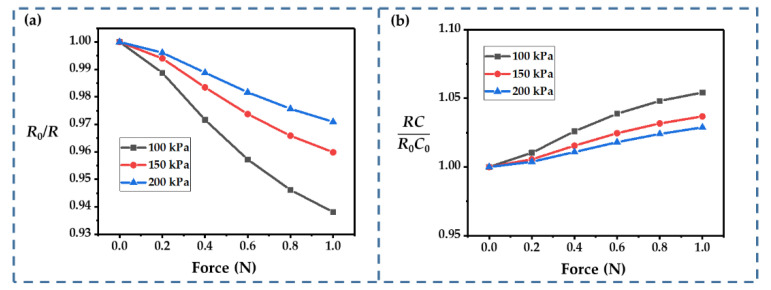
Influences of Young’s modulus of the electrolyte film on (**a**) *R*_0_/*R* and (**b**) *RC*/*R*_0_*C*_0_.

The Young’s modulus of the electrolyte film affects the displacement of the electrode under external forces, but it can hardly be controlled. However, since the Young’s modulus of the film increases with time, calculating the effect of the Young’s modulus of the electrolyte film could improve the closeness of the simulation to the real-world usage scenarios of our IDTSC sensor. As shown in Figure 10, changes in the resistance and *RC* decreased as the Young’s modulus increased, which could be attributed to the higher Young’s modulus restraining the movement of the electrodes. With an increase in the Young’s modulus, *R*_0_/*R* remained linear. Hence, a higher Young’s modulus seemed to improve the linearity. It could also improve the high-impact tolerance, which expanded the applicability of our IDTSC to high-*g* impact sensing.

According to the optimization results, in order to achieve better linearity for our IDTSC sensor, the suitable electrolyte thickness and gap size were 0.6 mm and 1.25 mm, respectively. Considering the size limitations, the optimum number of fingers and width were 10 and 3 mm, respectively. Furthermore, the fabricated IDTSC should be exposed to air for an additional 24 h to eliminate the extra solvent and enhance its Young’s modulus.

## 4. Fabrication and Electrochemical Analysis

**Figure 11:**
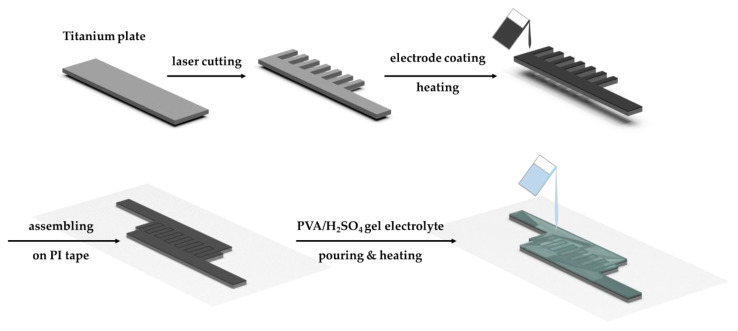
Illustration of the fabrication process of IDTSC.

The fabrication process of the IDTSC sensor is as follows (Figure 11): Firstly, a titanium metal plate is cut into a comb shape using high-precision laser cutting. A carbon/CMC/SBR slurry coating is then deposited onto the metal, which is left at 80 °C for 4 h to evaporate the solvent. Then, the prepared electrodes are transferred to the PI tape and assembled into an interdigital configuration. Finally, the H_2_SO_4_/PVA solution is poured onto the interdigital electrodes, which are heated at 60 °C until fully cured.

**Figure 12:**
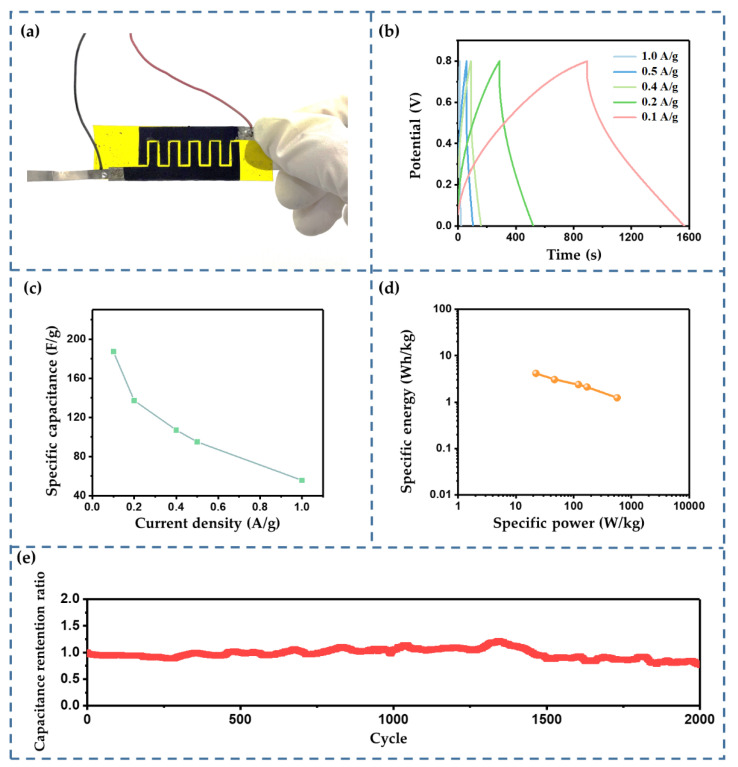
(**a**) The fabricated IDTSC sensor. (**b**) GCD curves of IDTSC at different current levels. (**c**) The specific capacitance vs. the current density. (**d**) Ragone plot of the IDTSC. (**e**) Cyclic durability characteristics of IDTSC.

Figure 12a shows the prepared IDTSC sensor, and its electrochemical performance was verified by the galvanostatic charge/discharge (GCD) method. As shown in Figure 12b, our IDTSC showed the necessary energy storage functions at different current levels (from 0.1 A/g to 1 A/g). The resistance of the IDTSC calculated from the IR drops ranged from 10 to 15 Ω, demonstrating that the device had a high resistance. This was acceptable, since proper resistance helps to improve sensitivity. Figure 12c shows the specific capacitance calculated from the GCD curves. The specific capacitance of the IDTSC ranged from 187.2 F/g to 55.7 F/g, corresponding to current density changes from 0.1 A/g to 1 A/g. As a visual display of the power supply performance, a Ragone plot was adopted to evaluate the output capability of the IDTSC. A Ragone plot is typically a log plot of power density versus corresponding energy density [28]. As shown in Figure 12d, the specific energy varied from 4.16 to 1.24 Wh/kg, and the specific power ranged from 22.26 to 566.06 W/kg. As shown in Figure 12e, the capacity retention was 78% after 2000 GCD cycles (at 1 A/g). These results showed that the IDTSC maintained the basic electrochemical properties of a supercapacitor and could be used as an energy device in real applications.

## 5. Sensing Behavior

**Figure 13:**
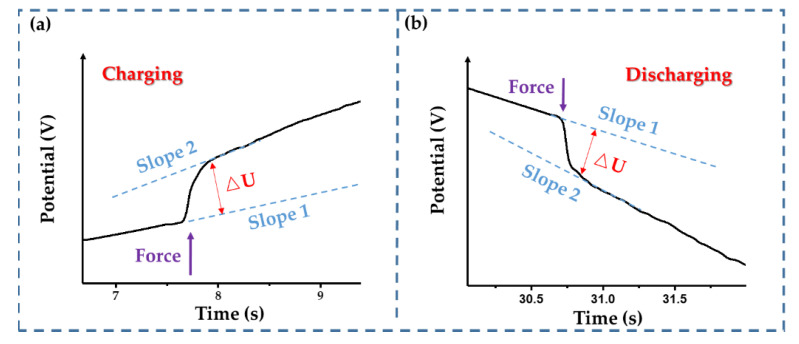
The potential changes while experiencing a step force during (**a**) the charging process and (**b**) the discharging process.

Firstly, the step-loading response of the IDTSC was tested. A step pressure was applied to the IDTSC in a GCD cycle. As shown in Figure 13, once the force was applied, there was a significant and rapid change in the device’s potential. The potential change was positive during charging and negative during discharging. Since the current directions during charging and discharging were opposite, the potential dominated by the resistance change was opposite. The transient potential rise and drop in Figure 13a,b proved that the resistance of the IDTSC increased under external forces, which was consistent with the simulation results. Furthermore, the slope of the curve increased after the force was applied, whether during charging or discharging.

For a supercapacitor, the change rate of the potential reflects its capacitance:

(10) $$i=C\frac{dU}{dt}$$

The increasing change rate of the potential indicated that the capacitance of our IDTSC decreased under external force, which was also consistent with the expected results.

**Figure 14:**
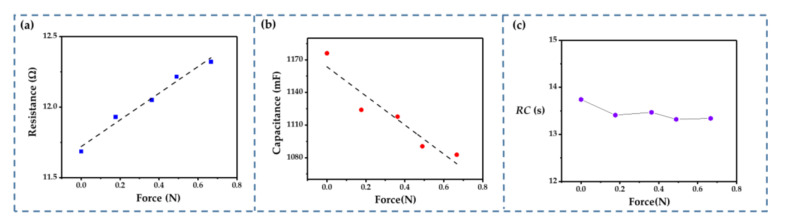
Test results: (**a**) resistance vs. external force. (**b**) capacitance vs. external force. (**c**) *RC* vs. external force.

The relationships between the resistance, the capacitance, and the applied force are shown in Figure 14a,b. The corresponding data were calculated from the GCD curve. Both the resistance and capacitance showed a good linear relationship in the given test range. The fabricated IDTSC had a good initial capacitance of 1170 mF and a suitable resistance of 11.7 Ω. The capacitance decreased to 1080 mF, and the internal resistance reached 12.3 Ω as the applied force reached 0.7 N. Furthermore, in agreement with the simulation optimization results, the *RC* of our IDTSC remained approximately stable as the force increased (Figure 14c).

**Figure 15:**
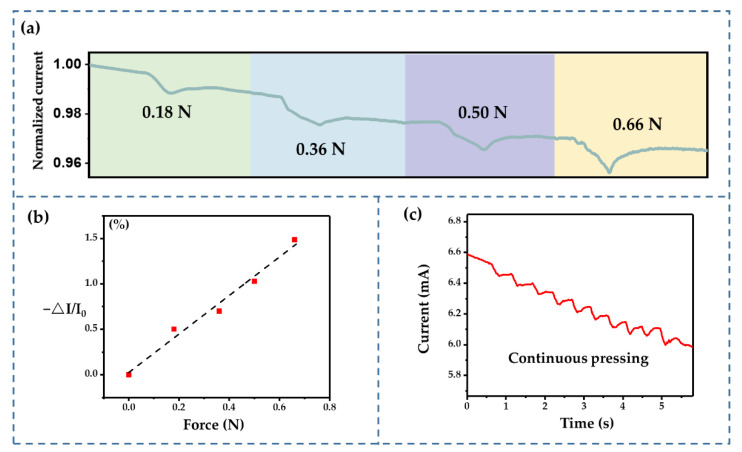
(**a**) The transient current response under different transient forces. (**b**) The current response increases with an increase in force. (**c**) Time domain current response under continuous pressing.

The resistance and capacitance vary under external force, which can result in current variation during constant voltage charging. Hence, the external force may be detected via transient fluctuations in the current. As shown in Figure 15a, the charging current dropped while a transient external force was applied—the greater the external force, the more significant the current drop. The change in the charging current varied linearly with the external force (Figure 15b), exhibiting good sensing properties and meeting our design goal. However, there was a deviation when substituting the actual resistance data into Equation (9). This was probably because the leakage current increased the total current, which is common in supercapacitors. As a result, the incorrectly estimated current increased the denominator in Equation (9), which may have caused an overestimation of the response sensitivity. The fabricated sensor also demonstrated its ability to sense continuous external forces (Figure 15c). The current varied under continuous pressing. The amplitude of these fluctuations was consistent, indicating that the sensing performance was stable.

**Figure 16:**
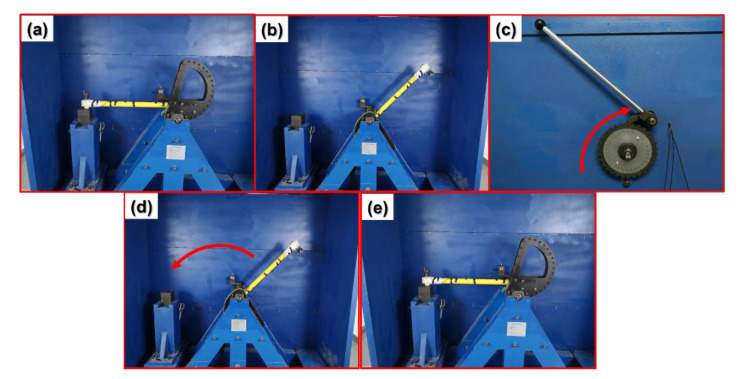
The machete hammer (**a**) used to generate high-g impact and its working process: (**b**) Lifting the hammerhead. (**c**) Pulling on the trigger. (**d**) Dropping the hammerhead. (**e**) Hitting the base to generate a high-*g* impact.

**Figure 17:**
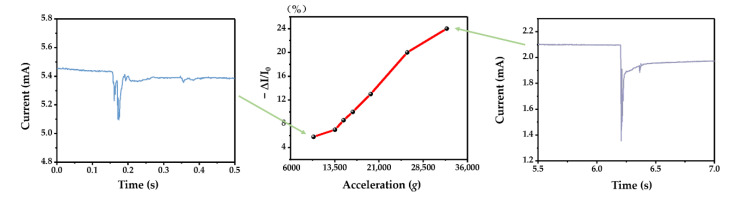
The current response changes according to acceleration increases.

Since the prepared IDTSC was lightweight and the gel electrolyte could provide reliable support, this sensor can be applied in high-*g* acceleration sensing. A machete hammer was used to generate a high-*g* impact on the device (Figure 16). As shown in Figure 17, the response increased with the acceleration from 9.9 × 10^3^× *g* to 3.2 × 10^4^× *g*. The change ratio is 6% at 9.9 × 10^3^× *g* and 24% at 3.2 × 10^4^× *g*. When the acceleration was high, the current showed relaxation, returning slowly after the impact. This suggested that the device may not be suitable for continuous high-*g* acceleration sensing, especially when the acceleration is high.

## 6. Conclusions

This article presented a force/acceleration sensor based on an interdigital supercapacitor. This device not only maintained the basic supercapacitor properties but also could be used to sense external forces through its corresponding transient current response. According to the theoretical and simulation analyses of the design, the current output of the device exhibited good linearity over a range of 0–0.8 N. The sensor could even be applied in high-*g* impact acceleration sensing, still demonstrating a responsive output at accelerations of up to 3.2 × 10^4^× *g*.

Based on the idea of empowering an energy storage device with sensing capabilities, in this study we fabricated an integrated device for energy supply and force sensing, showing the development prospects of multifunctional supercapacitors.

## Figures and Tables

**Table 1:** Key parameters used in the simulation.

Parameter	Value (Unit)
Young’s modulus of electrode	2 × 10^11^ (Pa)
Poisson’s ratio of electrode	0.29
Young’s modulus of electrolyte	1 × 10^5^ (Pa)
Poisson’s ratio of electrolyte	0.35
Conductivity of electrolyte	1 (S/m)
Thickness of electrode *h*_1_	0.5 (mm)
Thickness of electrolyte *h*_2_	0.2 (mm)
Gap between electrodes *d*	1.25 (mm)
Length of electrode *L*	15 (mm)
Width of electrode w	3.5 (mm)

## Data Availability

Not applicable.
